# Serum Myostatin Predicts the Risk of Hepatocellular Carcinoma in Patients with Alcoholic Cirrhosis: A Multicenter Study

**DOI:** 10.3390/cancers12113347

**Published:** 2020-11-12

**Authors:** Ji Hyun Kim, Seong Hee Kang, Minjong Lee, Gi Soo Youn, Tae Suk Kim, Baek Gyu Jun, Moon Young Kim, Young Don Kim, Gab Jin Cheon, Dong Joon Kim, Soon Koo Baik, Dae Hee Choi, Ki Tae Suk

**Affiliations:** 1 Department of Internal Medicine, Kangwon National University School of Medicine, Chuncheon 24289, Korea; kimjihyun81@knuh.or.kr (J.H.K.); greatstone@kangwon.ac.kr (T.S.K.); dhchoi-md@kangwon.ac.kr (D.H.C.); 2 Department of Internal Medicine, Wonju Severance Christian Hospital, Yonsei University Wonju College of Medicine, Wonju 26426, Korea; shkang14@yonsei.ac.kr (S.H.K.); drkimmy@yonsei.ac.kr (M.Y.K.); baiksk@yonsei.ac.kr (S.K.B.); 3 Department of Internal Medicine, Ewha Womans University College of Medicine, Seoul 07804, Korea; 4 Department of Internal Medicine, Chuncheon Sacred Heart Hospital, Hallym University College of Medicine, Chuncheon 24253, Korea; 41641@hallym.ac.kr (G.S.Y.); djkim@hallym.ac.kr (D.J.K.); 5 Department of Internal Medicine, Gangneung Asan Hospital, University of Ulsan College of Medicine, Gangneung 25440, Korea; backyou78@ulsan.ac.kr (B.G.J.); ydkim@gnah.co.kr (Y.D.K.); 1000@gnah.co.kr (G.J.C.)

**Keywords:** myostatin, hepatocellular carcinoma, cirrhosis

## Abstract

**Simple Summary:**

Sarcopenia is prevalent in patients with liver cirrhosis. It has been well-known that cirrhotic patients with sarcopenia had poorer survival as compared to those without it, and expression of serum myostatin is associated with sarcopenia. In this study, we aimed to evaluate whether serum myostatin is associated with hepatocellular carcinoma development in patients with alcoholic cirrhosis or not. We found that serum myostatin is associated with hepatocellular carcinoma development in a large cohort of patients with alcoholic cirrhosis. Among patients with similar residual liver function or similar risk of developing hepatocellular carcinoma using other models, higher serum myostatin was significantly associated with a higher risk of developing hepatocellular carcinoma. In addition, patients with more advanced hepatic fibrosis had significantly higher serum myostatin than those with less advanced hepatic fibrosis. Thus, serum myostatin as a prognostic marker can be useful to identify high-risk patients who need stringent surveillance for hepatocellular carcinoma.

**Abstract:**

*Background and Aim:* Previous studies reported that serum myostatin is associated with sarcopenia. We aimed to elucidate the association between serum myostatin levels and hepatocellular carcinoma (HCC) development in patients with alcoholic liver cirrhosis (ALC). *Methods:* This retrospective, multicenter study assessed 1077 Asian ALC patients enrolled from 2007 to 2017. The primary endpoint was the development of HCC within 5 years. Cox proportional hazards model analyses were used to assess the association of serum myostatin levels and HCC development. The time-dependent areas under the receiver operating characteristic curve (AUROC) of serum myostatin for 5-year HCC development were calculated. Serum myostatin levels were measured using an enzyme-linked immunosorbent assay with samples collected on the index date. *Results:* During a median follow-up of 2.5 years, 5-year cumulative HCC incidence rates were 6.7% in the total population. The median level of serum myostatin was 3.3 ng/mL (interquartile, 2.1–5.2 ng/mL). The AUROC of serum myostatin for 5-year HCC development was 0.78 (95% confidence interval [CI], 0.76–0.81). In Cox proportional hazards model analyses, age, gender, platelet counts, and serum myostatin levels were independent risk factors for HCC development (adjusted hazard ratios [HRs] of age, male gender, platelet counts, and serum myostatin: 1.03, 2.79, 0.996, 1.18, respectively; all *p* < 0.05). Patients with high myostatin levels had a significantly higher risk of 5-year HCC development than those with low myostatin levels (HR 7.53, *p* < 0.001). *Conclusion:* Higher serum myostatin levels were significantly associated with a higher risk of developing HCC in ALC patients, which could identify high-risk patients who need stringent surveillance.

## 1. Introduction

Sarcopenia is a syndrome characterized by age-related skeletal muscle loss, which is prevalent in adults with cancer and chronic comorbidities such as liver cirrhosis [1,2,3]. The prevalence of sarcopenia in patients with cirrhosis is reported to be >40% [4,5]. Several studies have reported that sarcopenia is associated with the development and recurrence of hepatocellular carcinoma (HCC) [6,7].

Recently, it was reported that higher serum myostatin levels were significantly correlated with muscle mass loss, hyperammonemia, and impaired protein synthesis in cirrhotic patients [8,9]. It was also suggested that serum myostatin levels represented a promising factor for the prediction of clinical outcomes in cirrhotic patients [9]. In addition, preclinical studies showed that myostatin expression was significantly increased in different tumor tissues and could block cancer cell death by regulating mitochondrial metabolism [10]. Myostatin aggravates hepatic fibrosis through activation of hepatic stellate cells via c-Jun n-terminal kinase activation [11].

Previous studies have reported that serum myostatin levels are significantly elevated in patients with end-stage liver disease or sarcopenia, and are associated with poorer survival in cirrhotic patients compared with those who had low serum myostatin levels [9,12,13]. In previous studies performed in cirrhotic patients with chronic hepatitis B or C, serum myostatin showed a significant association with survival and HCC development [9,14]. However, there was a lack of study to evaluate association between serum myostatin and clinical outcomes in patients with alcoholic cirrhosis. Particularly, whether serum myostatin levels are associated with HCC development in patients with alcoholic cirrhosis remains unsolved. A new prognostic marker for HCC development could help to monitor high-risk cirrhotic patients. The aim of this study was to investigate the prognostic performance of serum myostatin levels on HCC development in patients with alcoholic liver cirrhosis (ALC).

## 2. Methods

### 2.1. Patients

This was a retrospective study using prospective databases. Patients were consecutively admitted to 3 tertiary University hospitals in Korea (Kangwon National University Hospital, Wonju Severance Christian Hospital, Chuncheon Sacred Heart Hospital) between January 2007 and December 2017. A total of 1077 ALC patients, who had stored sera available to measure myostatin levels, were included. Serum samples were collected from 3 previous liver cirrhosis studies (cris.nih.go.kr, registration nos. KCT0003801 and KCT0000257; clinicaltrials.gov, registration no. NCT04339725). Serum myostatin was measured using commercially available kits (R&D Systems, Minneapolis, MN, USA) at one center (Chuncheon Sacred Heart Hospital) after collecting samples from all three centers. All included patients who had alcoholic cirrhosis. Patients were excluded if they met any of the following criteria: Active alcoholism; younger than 18 years or older than 85 years; a diagnosis of HCC before study enrollment; infection with hepatotropic viruses (i.e., hepatitis B, C, or D viruses) or human immunodeficiency virus; and liver transplantation. The following criteria determined a clinical diagnosis of ALC: (i) A history of alcohol intake (daily alcohol intake > 80 g for men and > 60 g for women for greater than 5 years); (ii) platelet counts < 150,000/mL and ultrasonography findings suggestive of cirrhosis, including blunted, nodular liver edges accompanied by splenomegaly (>12 cm); or (iii) clinical signs of portal hypertension, such as ascites, esophageal, or gastric varices, and hepatic encephalopathy [15,16,17,18,19,20]. An HCC diagnosis was based on the radiologic findings of dynamic computed tomography (CT) and/or magnetic resonance imaging (MRI), which included intense arterial uptake followed by a contrast washout during the venous-delayed phases. For patients who had equivocal findings on CT, MRI with liver-specific contrast was performed to diagnose HCC. If characteristic findings of HCC on MRI were not found, a liver biopsy was performed [21,22,23,24,25,26]. The study protocol complied with the ethical guidelines of the World Medical Association Declaration of Helsinki and was approved by the Institutional Review Board of each hospital.

### 2.2. Clinical Evaluation and Follow-Up

The primary outcome was HCC development within 5 years. The index date was defined as the date of blood sampling that included serum myostatin levels when patients were admitted to each hospital to evaluate cirrhotic statuses or complications associated with cirrhosis. Follow-up was considered the time interval between the index date and the last available clinical information: The date of an HCC diagnosis or the last follow-up visit without HCC development. Regular follow-up observations included blood tests and radiological assessments by ultrasonography, CT, or MRI every 3–6 months to detect HCC incidences.

Hepatic decompensation was defined as variceal bleeding, ascites, hepatic encephalopathy, or hepatorenal syndrome; in the case of patients with multiple hepatic decompensation events, the patient was regarded as having “one” hepatic decompensation as an outcome and the earliest decompensation was used for the statistical analysis.

### 2.3. Statistical Analysis

The data were presented as the median with an interquartile range (IQR) for continuous variables and numbers and percentages for categorical variables. Continuous levels of serum myostatin were divided into two groups, the low myostatin and high myostatin groups, using median values that were then treated as dichotomous covariates for Kaplan–Meier curves analyses. The Mann–Whitney U and Kruskal–Wallis tests were used to analyze differences between the different groups. The χ^2^ test or Fisher exact test was used for categorical data.

The development of HCC was analyzed with Kaplan–Meier curves and compared using the log-rank test. Variables with a *p*-value < 0.05 in our univariate analyses were finally entered into multivariate analyses using the Cox proportional hazard model. Time-dependent areas under receiver operating characteristic (AUROC) curves for censored data were constructed to define the best cutoff value for predicting HCC development within five years [27]. The best cutoff value was defined as the value with a maximal sum of the sensitivity and specificity that could predict HCC development within 5 years [28]. Confidence intervals (CI) for AUROC were obtained from 1000 bootstrapped samples. A *p*-value < 0.05 was considered to be statistically significant. All statistical analyses were conducted using the R statistical programming environment (v3.5.1; http://www.r-project.org).

## 3. Results

### 3.1. Baseline Characteristics

A total of 1077 cirrhotic patients were included in this study. The baseline characteristics are listed in Table 1. Our study cohort included 84.4% males with a median age of 55 years, and 31.8%, 43.1%, and 25.1% were in Child–Pugh class A, B, and C, respectively. Patients diagnosed with HCC were older (57 years vs. 55 years; *p* = 0.003) and had a higher proportion of male patients (94.9% vs. 82.2%; *p* = 0.002), higher body mass indices (BMIs; 24.1 vs. 22.9; *p* = 0.009), lower median platelet counts (93 vs. 107 × 10^9^/L; *p* = 0.03), higher Toronto HCC risk index (THRI; 255 vs. 236; *p* < 0.001), higher fibrosis-4 indices (FIB-4; 8.0 vs. 6.4; *p* = 0.03), and higher serum myostatin levels (6.1 vs. 3.2 ng/mL; *p* < 0.001) compared with those without HCC. The median myostatin levels were 3.3 ng/mL in the total population, 3.2 ng/mL among patients without HCC, and 6.1 ng/mL among patients with HCC (*p <* 0.001).

### 3.2. Serum Myostatin Levels as an Independent Prognostic Factor for the Development of Hepatocellular Carcinoma within 5 Years

**Figure 1:**
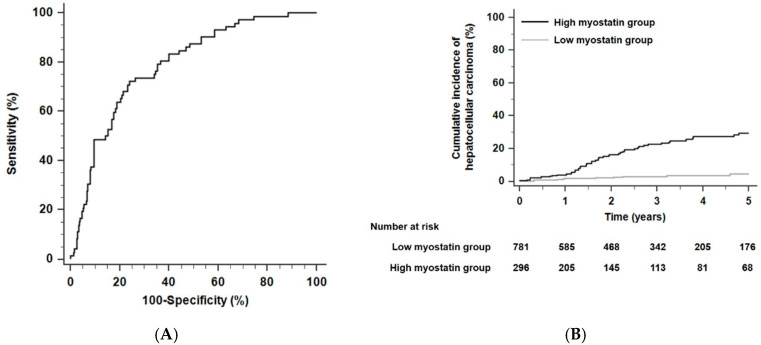
The predictive performance of serum myostatin levels and the cumulative probability of HCC in alcoholic liver cirrhosis (ALC) patients. (**A**) Time-dependent areas under receiver operating characteristic curves (AUROCs) of serum myostatin to predict HCC development within five years. (**B**) Risk stratification for HCC development in ALC patients according to serum myostatin levels (*p* < 0.001 by log-rank test). Abbreviations: ALC, alcoholic liver cirrhosis; AUROC, area under the receiver operating characteristic curve; HCC, hepatocellular carcinoma.

Regarding the performance of predicting 5-year HCC risk, the time-dependent AUROC of serum myostatin was 0.78 (sensitivity, 72.3%; specificity, 72.1%; positive predictive value 25.2%; negative predictive value 95.3%; 95% CI, 0.71–0.84; Figure 1A) and the time-dependent AUROC of serum myostatin for 3-year HCC risk was 0.79 (sensitivity, 74.7%; specificity, 75.2%; positive predictive value 20.8%; negative predictive value 97.2%; 95% CI, 0.72–0.85). The total population was divided into two groups (a low myostatin group and a high myostatin group) using a cutoff point of 4.97 ng/mL, which provided the maximum sum of the specificity and sensitivity in being able to predict the risk of HCC development within 5 years. Patients with low serum myostatin levels were classified into the low myostatin group (<4.97 ng/mL, *n* = 781), and those with high serum myostatin levels were classified into the high myostatin group (≥4.97 ng/mL, *n* = 296).

Over a 2.5-year median follow-up period (interquartile range [IQR], 1.2–4.5 years), the 5-year HCC incidence rates were 17.6% in the high myostatin group (*n* = 52) and 2.6% in the low myostatin group (*n* = 20) (hazard ratio [HR] 7.51, 95% CI 4.43–12.75, *p <* 0.001 by the log-rank test; Figure 1B). In the competing risk analysis, patients with high serum myostatin also had a significantly higher risk of HCC development than those with low serum myostatin (HR 6.80, 95% CI 4.20–10.97, *p <* 0.001). For the multivariate Cox analyses, age, gender, platelet counts, and serum myostatin levels were found to be independent risk factors for HCC development (HR for age 1.03, 95% CI 1.01–1.06, *p* = 0.004; HR for gender 2.79, 95% CI 1.01–7.75, *p* = 0.04; HR for platelet counts 0.996, 95% CI 0.992–0.999, *p* = 0.03; and HR for serum myostatin 1.18, 95% CI 1.12–1.24, *p* < 0.001; Table 2).

### 3.3. The Prognostic Performance of Serum Myostatin to Predict HCC Risk in Different Risk Groups According to the Toronto HCC Risk Index

**Figure 2:**
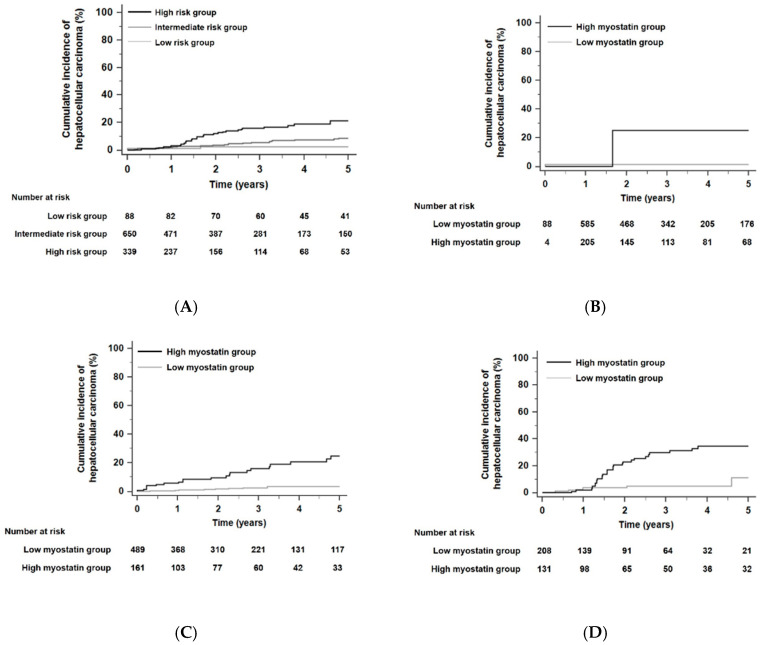
Kaplan–Meier estimates of HCC development in ALC patients according to the Toronto HCC risk index (THRI). (**A**) Risk stratification for HCC development in ALC patients according to the THRI (*p* < 0.001 by log-rank test). (**B**) Risk stratification for HCC development according to serum myostatin levels in the low risk group stratified by the THRI; (**C**) in the intermediate risk group; (**D**) in the high-risk group (all, *p* < 0.05 by log-rank test). Abbreviations: ALC, alcoholic liver cirrhosis; THRI, Toronto HCC risk index; HCC, hepatocellular carcinoma.

When the HCC risk of all patients was stratified according to the THRI [29], the 5-year risk of developing HCC was significantly different among the different risk groups (*p* < 0.001 by the log-rank test, Figure 2A): HCC developed within 5 years in 2.3% of patients in the low-risk group (*n* = 88), 4.9% of patients in the intermediate-risk group (*n* = 650), and 11.2% of patients in the high-risk group (*n* = 339) according to the THRI stratification. The time-dependent AUROC of the THRI for 5-year HCC risk was 0.72 (95% CI, 0.65–0.78). In each risk group, HCC risk was significantly different according to serum myostatin levels. In each risk group stratified by the THRI, patients with high myostatin levels had significantly higher HCC risk than those with low myostatin levels. The HR was 18.89 in the low-risk group with a 95% CI of 1.18–303.25 (*p* = 0.04, Figure 2B), the HR was 6.65 in the intermediate-risk group with a 95% CI of 3.38–13.08 (*p* < 0.001, Figure 2C), and the HR was 4.01 in the high-risk group with a 95% CI of 1.96–8.22 (*p* < 0.001, Figure 2D). In competing risk analyses, patients with high myostatin levels also had significantly higher HCC risk than those with low myostatin levels in each risk group stratified by the THRI. The HR was 19.89 in the low-risk group with a 95% CI of 1.59–248.50 (*p* = 0.02), the HR was 5.85 in the intermediate-risk group with a 95% CI of 2.99–11.45 (*p* < 0.001), and the HR was 4.41 in the high-risk group with a 95% CI of 2.12–9.16 (*p* < 0.001).

### 3.4. Subgroup Analyses According to Age, Sex, and Child–Pugh Class

Predictive performance of serum myostatin levels for development of HCC was significantly different according to age (≥65, < 65 years old), sex, and Child–Pugh class. The predictive performance of serum myostatin levels for the development of HCC was higher in patients with age < 65 years old than that in patients with age ≥ 65 years old: Time-dependent AUROC of serum myostatin levels for development of HCC within 5 years was 0.81 (95% confidence interval [CI] 0.77–0.83) in those with age < 65 years old (*n* = 874) and 0.68 (95% CI 0.61–0.74) in patients with age ≥ 65 years old (*n* = 203). Predictive performance of serum myostatin levels for development of HCC was higher in male patients than that in female patients: Time-dependent AUROC of serum myostatin levels for development of HCC within 5 years was 0.77 (95% CI 0.74–0.80) in male patients (*n* = 895) and 0.62 (95% CI 0.55–0.69) in female patients (*n* = 182). Predictive performance of serum myostatin levels for development of HCC was higher in patients with Child–Pugh class A than that in those with Child–Pugh B and C: Time-dependent AUROC of serum myostatin levels for development of HCC within 5 years was 0.80 (95% CI 0.75–0.84) in patients with Child–Pugh class A (*n* = 343), 0.77 (95% CI 0.73–0.81) in patients with Child–Pugh class B (*n* = 464), and 0.75 (95% CI 0.70–0.80) in patients with Child–Pugh class C (*n* = 270). Predictive performance of serum myostatin levels for development of HCC was higher in patients with age < 65 years old, male, and Child–Pugh class A as compared to those with age ≥ 65 years old, female, and Child–Pugh class B/C.

### 3.5. The Prognostic Performance of Serum Myostatin Levels to Predict HCC Risk According to Residual Functional Reserves and Hepatic Fibrosis

**Figure 3:**
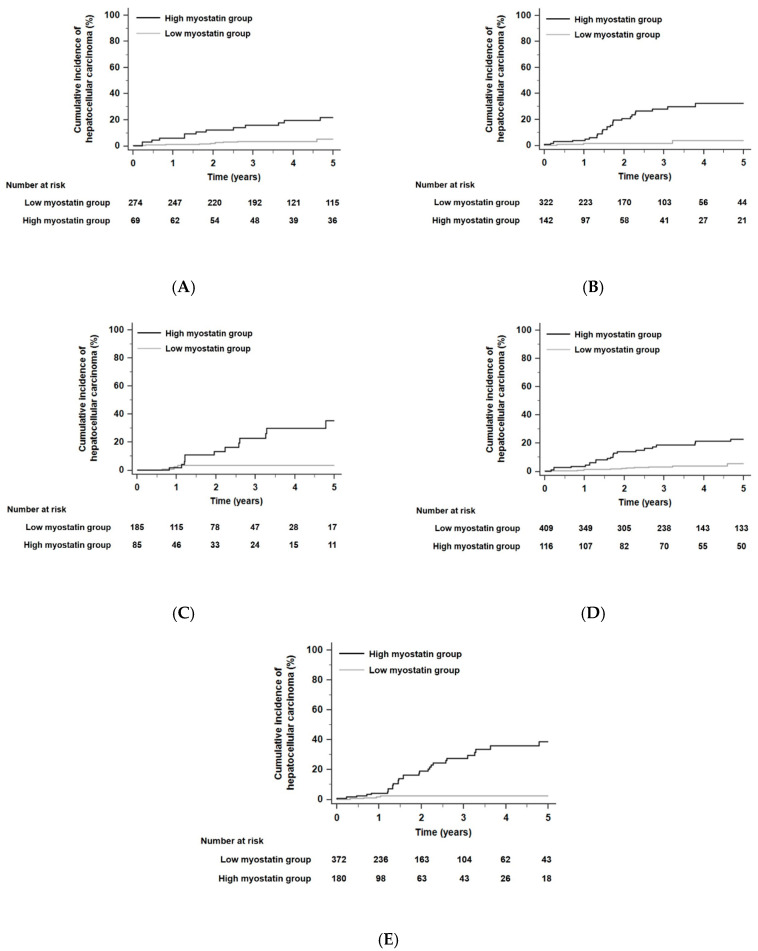
Kaplan–Meier estimates of HCC development in ALC patients in each group who had similar residual liver function according to Child–Pugh class or model for end-stage liver disease (MELD) scores. Risk stratification for HCC development according to serum myostatin levels among patients who were in Child–Pugh class A (**A**), class B (**B**), and class C (**C**) (all, *p* < 0.001 by log-rank test). Risk stratification for HCC development according to serum myostatin levels among patients who had low MELD scores (≤13, median value) (**D**) and high MELD scores (>13) (**E**) (all, *p* < 0.001 by log-rank test). Abbreviations: ALC, alcoholic liver cirrhosis; HCC, hepatocellular carcinoma; MELD, model for end-stage liver disease.

When the residual liver function of all patients was stratified according to Child–Pugh class, the 5-year risk of developing HCC was not significantly different in each Child–Pugh class (*p* = 0.13 by the log-rank test): HCC developed within 5 years in 6.7% of patients in Child–Pugh class A (*n* = 343), 7.1% of patients in Child–Pugh class B (*n* = 464), and 5.9% of patients in Child–Pugh class C (*n* = 270). HCC risks as predicted by serum myostatin levels were significantly different for each Child–Pugh class. In each Child–Pugh class, patients with high myostatin levels had higher HCC risks than those with low myostatin levels. The HR was 4.94 in Child–Pugh class A patients with a 95% CI of 1.81–13.52 (*p* < 0.001, Figure 3A), the HR was 11.05 in Child–Pugh class B patients with a 95% CI of 5.21–23.46 (*p* < 0.001, Figure 3B), and the HR was 6.55 in Child–Pugh class C patients with a 95% CI of 2.28–18.83 (*p* < 0.001, Figure 3C). In competing risk analyses, patients with high myostatin levels also had higher HCC risks than those with low myostatin levels in each Child–Pugh class. The HR was 4.50 in Child–Pugh class A patients with a 95% CI of 2.02–10.04 (*p* < 0.001), the HR was 8.05 in Child–Pugh class B patients with a 95% CI of 3.80–17.02 (*p* < 0.001), and the HR was 6.52 in Child–Pugh class C patients with a 95% CI of 2.10–20.20 (*p* < 0.001).

When the total patient population of this study was divided according to a median model for end-stage liver disease (MELD) score of 13, the 5-year risk of developing HCC was not significantly different according to the low and high MELD score groups (*p* = 0.05 by the log-rank test): HCC developed within 5 years in 6.9% of patients in the low MELD scores group (*n* = 525) and 6.5% of patients in the high MELD scores group (*n* = 552). In each MELD score group, patients with high myostatin levels had higher HCC risks compared with those with low myostatin levels. The HR was 6.39 among patients with low MELD scores (≤13) with a 95% CI of 3.10–13.20 (*p* < 0.001, Figure 3D), and the HR was 9.58 among patients with high MELD scores (>13) with a 95% CI of 4.45–20.61 (*p* < 0.001, Figure 3E). In competing risk analyses, patients with high myostatin levels also had higher HCC risks compared with those with low myostatin levels in each MELD score group. The HR was 5.36 among patients with low MELD scores (≤13) with a 95% CI of 2.85–10.07 (*p* < 0.001), and the HR was 8.02 among patients with high MELD scores (>13) with a 95% CI of 3.64–17.64 (*p* < 0.001).

**Figure 4:**
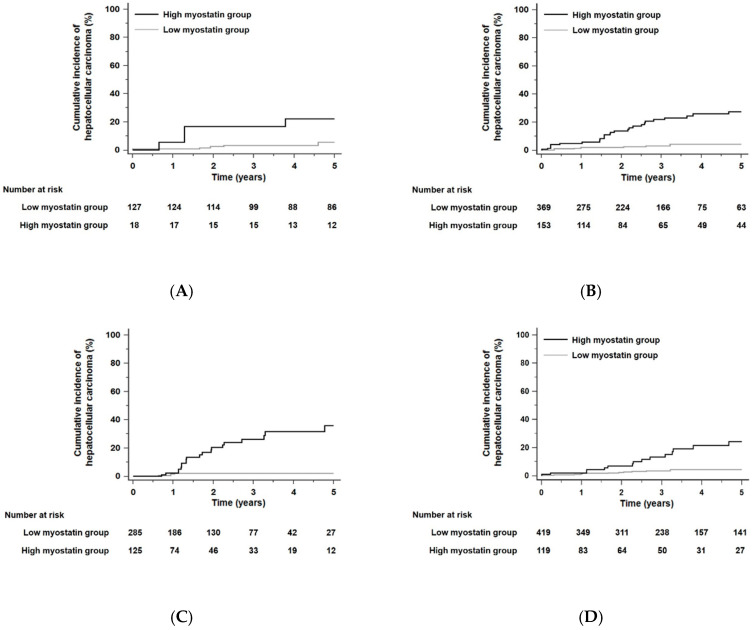

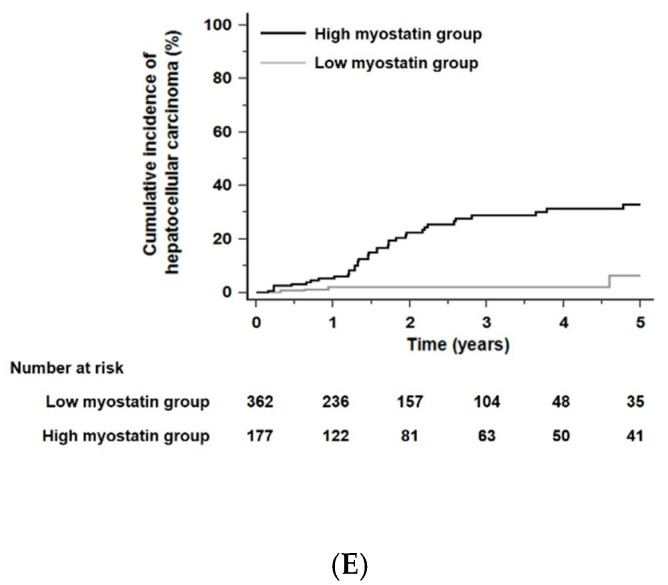
Kaplan–Meier estimates of HCC development in ALC patients in each group who had similar residual liver function according to the albumin–bilirubin (ALBI) grade or fibrosis-4 (FIB-4) scores. Risk stratification for HCC development according to serum myostatin levels among patients who were in ALBI grade 1 (**A**), grade 2 (**B**), and grade 3 (**C**) (all *p* < 0.01 by log-rank test). Risk stratification for HCC development according to serum myostatin levels among patients who had low FIB-4 scores (≤6.46, median value) (**D**) and high FIB-4 scores (>6.46) (**E**) (all *p* < 0.001 by log-rank test). Abbreviations: ALBI, albumin–bilirubin grade; ALC, alcoholic liver cirrhosis; FIB-4, fibrosis-4; HCC, hepatocellular carcinoma.

When the residual liver function of all patients was stratified according to the albumin–bilirubin (ALBI) grade [30], the 5-year risk of developing HCC was not significantly different in each ALBI grade (*p* = 0.30 by the log-rank test): HCC developed within 5 years in 6.9% of patients in ALBI grade 1 (*n* = 145), 7.3% of patients in ALBI grade 2 (*n* = 522), and 5.9% of patients in ALBI grade 3 (*n* = 410). HCC risks as predicted by serum myostatin levels were significantly different for each ALBI grade. In each ALBI grade, patients with high serum myostatin levels had higher HCC risks than those with low serum myostatin levels. The HR was 4.81 in ALBI grade 1 patients with a 95% CI of 1.36–16.95 (*p* = 0.03, Figure 4A), the HR was 6.76 in ALBI grade 2 patients with a 95% CI of 3.29–13.88 (*p* < 0.001, Figure 4B), and the HR was 12.51 in ALBI grade 3 patients with a 95% CI of 4.30–36.44 (*p* < 0.001, Figure 4C).

When the total patient population of this study was divided according to a median fibrosis-4 (FIB-4) score [31] of 6.46, the 5-year risk of developing HCC in patients with a high FIB-4 score (>6.46) was significantly higher than that in those with a low FIB-4 score (≤6.46; HR = 2.20, 95% CI = 1.37–3.53, *p* = 0.001): HCC developed within 5 years in 5.2% of patients in the low FIB-4 scores group (*n* = 538) and 8.2% of patients in the high FIB-4 scores group (*n* = 539). Irrespective of low or high FIB-4 score groups, patients with high serum myostatin levels had higher HCC risks compared with those with low serum myostatin levels. The HR was 5.18 among patients with low FIB-4 scores (≤6.46) with a 95% CI of 2.47–10.85 (*p* < 0.001, Figure 4D), and the HR was 9.53 among patients with high FIB-4 scores (>6.46) with a 95% CI of 4.26–21.33 (*p* < 0.001, Figure 4E).

### 3.6. Correlation between Serum Myostatin Levels and Hepatic Fibrosis

Regarding hepatic fibrosis, we evaluated the correlation between serum myostatin levels and hepatic fibrosis using the FIB-4 scores [31]. Hepatic fibrosis was graded into 3 categories: Group 1 with FIB-4 score < 1.45, group 2 with FIB-4 score between 1.45 and 3.25, and group 3 score with FIB-4 > 3.25 [31]. At the time of measurement of serum myostatin levels, median serum myostatin levels were increased as FIB-4 scores were higher: Median serum myostatin levels were 1.0 ng/mL in group 1, 2.5 ng/mL in group 2, and 3.5 ng/mL in group 3, respectively. There was a significant correlation between FIB-4 groups and serum myostatin levels (*Pearson*’s correlation coefficient = 0.33, *p* < 0.001): Patients in higher FIB-4 groups had higher serum myostatin levels. Follow-up data of FIB-4 scores at 3 years were also evaluated. Patients were categorized into two groups: Patients with FIB-4 scores > 3.25 and ≤ 3.25 at 3 years. Patients with higher FIB-4 scores had significantly higher serum myostatin levels of 3.8 ng/mL than 3.0 ng/mL in those with lower FIB-4 scores (*p* < 0.001).

### 3.7. Distant Metastasis, Serum α-Fetoprotein, and Serum Myostatin Levels

Serum myostatin levels were not significantly associated with distant metastasis in patients with HCC (*n* = 79). Among HCC patients (*n* = 79), only two HCC patients with distant metastasis showed a median value of serum myostatin level 6.8 ng/mL: A median value of serum myostatin level was also 6.8 ng/mL in HCC patients without distant metastasis. When HCC patients were divided into two groups (patients with HCC within early stage [*n* = 62] vs. patients with HCC beyond early stage [*n* = 17] based on the Barcelona Clinic Liver Cancer [BCLC] staging system), a median serum myostatin level of 6.7 ng/mL in patients with HCC within early stage was not significantly different from that of 7.2 ng/mL in patients with HCC beyond the early stage (*p* = 0.74)

We compared the prognostic performance of serum alpha-fetoprotein (AFP) with that of serum myostatin. Time-dependent areas under receiver operating characteristic (AUROC) curve of serum AFP levels to predict development of HCC were significantly lower than those of serum myostatin (*p* < 0.001 by DeLong test): Serum AFP, 0.57 (95% confidence interval [CI], 0.54–0.60) and serum myostatin, 0.78 (95% CI, 0.71–0.84), respectively. Serum AFP levels just before the diagnosis of HCC were also evaluated and the median value of serum AFP levels were 4.3 ng/mL with interquartile range of 2.4–10.7 ng/mL. There was no significant difference of serum AFP levels between those with HCC and without HCC. Patients were divided into two groups according to serum AFP levels (median value): Patients with low serum AFP levels (<4 ng/mL) and those with high serum AFP levels (≥4 ng/mL). In patients with high serum AFP levels, patients with high serum myostatin levels had a significantly higher HCC risk than those with low serum myostatin levels (HR 5.22, 95% CI 2.77–9.86, *p* < 0.001). In patients with low serum AFP levels, patients with high serum myostatin levels had a significantly higher HCC risk than those with low serum myostatin levels (HR 9.12, 95% CI 4.25–19.57, *p* < 0.001). Patients with both high serum myostatin and high serum AFP levels had 5-year HCC risks up to 18.0%, a significantly higher risk of HCC development than 1.4%, 5-year HCC risk with both low serum myostatin and low serum AFP levels (HR 14.70, 95% CI 5.72–37.81, *p* < 0.001).

### 3.8. Risk Assessments for Developing Hepatic Decompensation and Liver-Related Death According to Serum Myostatin Levels

**Figure 5:**
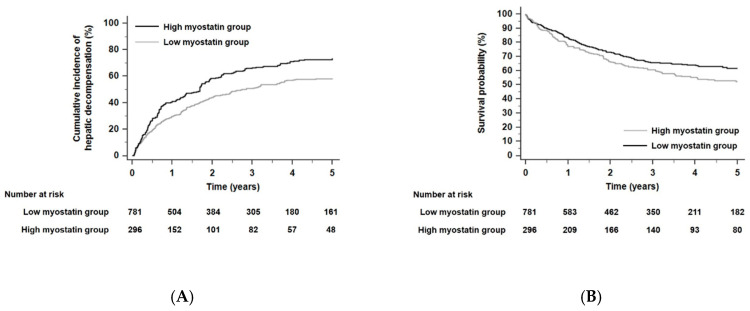
Kaplan–Meier estimates of hepatic decompensation event (**A**) and liver-related death (**B**) in ALC patients according to serum myostatin levels (all *p* < 0.001 by log-rank test). Abbreviations: ALC, alcoholic liver cirrhosis.

The cumulative incidence rates with developing any hepatic decompensation event or liver-related death within 5 years were 54.5% (*n* = 587) and 34.8% (*n* = 375), respectively. Patients in the high myostatin group were more likely to develop any hepatic decompensation event than those in the low myostatin group, who were used as a reference. (HR 1.47, 95% CI 1.23–1.75, *p* < 0.001, Figure 5A). Similarly, the risk of liver-related death significantly increased in the high myostatin group compared with the low myostatin group (HR 1.34, 95% CI 1.12–1.62, *p* = 0.02, Figure 5B).

## 4. Discussion

This study showed the prognostic performance of serum myostatin for HCC development and also validated the influence of serum myostatin levels on survival in a previous study in a large cohort of patients with cirrhosis [9]. The present study showed that high baseline serum myostatin levels were significantly associated with a higher risk of developing HCC within 5 years in ALC patients. Among patients with similar HCC risks stratified by THRI, patients with high myostatin had significantly higher risks of developing HCC than those with low myostatin. In addition, within the same Child–Pugh class, the ALBI grade, or similar MELD score, which reflects residual functional reserves, HCC risk was significantly different according to serum myostatin levels. Furthermore, elevated serum myostatin levels were associated with a higher risk of hepatic decompensation and poor risk for survival in ALC patients.

In this study, we have shown that serum myostatin could be a prognostic factor for HCC development in ALC patients. The possible mechanism of high myostatin levels being significantly associated with HCC development can be explained by a direct link between myostatin and hepatic fibrogenesis. Myostatin activates a fibrotic progression via C-Jun N-terminal kinase activation or NF-κB-dependent activation: The muscle-liver axis (Supplementary Figure S1) [11,32,33,34,35]. Given that ongoing hepatic fibrosis is an important factor for HCC development [36], high myostatin levels in high-risk patients activate fibrotic progression and likely play a role in HCC development. Myostatin can be also directly involved in HCC carcinogenesis. Previous studies have reported that myostatin expression is significantly increased in different human cancer tissues and cells, and that myostatin knockdown increases cancer cell apoptosis via modulation of mitochondrial metabolism and reactive oxygen species [10,37]. It has been also shown that inactivation of the myostatin gene decreases tumor growth in myostatin-knockout animal models [38].

In our study, a one-point increase in serum myostatin levels was associated with a 1.2-fold increase in the risk of developing HCC. Serum myostatin at the baseline stratified the HCC risk among patients with similar risks of HCC development based on the THRI [29,39] or similar residual liver function using Child–Pugh classifications. Particularly, in the low-risk group stratified with the THRI, patients with high serum myostatin levels had significantly higher 5-year HCC risks than those with low serum myostatin levels. Similarly, among patients with Child–Pugh class A, who had good residual liver function, those with high serum myostatin levels had higher 5-year HCC risks than those with low serum myostatin levels. Patients with high serum myostatin levels in the high-risk group based on the THRI stratification had high 5-year HCC risks up to 22.1%. It would be possible to more accurately identify high-risk patients using baseline myostatin levels in patients who have similar residual liver functions or HCC risks based on other methods, which would thereby guide physicians to plan surveillance strategies for patients at risk of developing HCC.

Although serum AFP has been a well-known marker for HCC development, the predictive performance of serum AFP for HCC development in this study was not high. The reason may be the small number of patients who developed HCC (*n* = 79), or that HCC in most patients was diagnosed within the early stage. When the combination of serum AFP and serum myostatin levels was used, patients with both high serum myostatin and high serum AFP levels had a significantly higher risk of HCC development than those with both low serum myostatin and low serum AFP levels. The two markers can be helpful to identify high-risk patients for HCC development and guide physicians to perform strict HCC surveillance.

The present study found that serum myostatin levels were also useful in stratifying the risk of hepatic decompensation and liver-related deaths. Our results support that serum myostatin levels provide a significant prognostic value for determining long-term clinical outcomes in ALC patients. Considering that patients with high serum myostatin levels at the baseline had higher risks of HCC, hepatic decompensation, and liver-related death than those with low myostatin levels, myostatin might be more involved with fibrotic progression than carcinogenesis in the liver. In other words, myostatin expression could be one treatment target to prevent sarcopenia and hepatic fibrosis, thereby lowering the risk of hepatic decompensation and HCC development.

There were several limitations to this study. First, it was a limitation that serum myostatin levels in cirrhotic patients with other etiologies of non-alcoholic steatohepatitis (NASH) or chronic viral hepatitis were not evaluated in this study. Although we could not analyze serum myostatin levels in cirrhotic patients with chronic hepatitis B or C because of no available serum, we guess that serum myostatin levels in patients with alcoholic cirrhosis may be similar to those with chronic viral hepatitis: Median levels of serum myostatin were 3.3 ng/mL in our population with alcoholic cirrhosis and 3.1 ng/mL in patients who mainly had etiologies of chronic viral hepatitis B or C in a previous study [9]. Previous studies showed that serum myostatin also had good prognostic performance of survival and HCC development in a population of whom 58.6% had predominantly hepatitis C virus (HCV)-related liver cirrhosis [9] and 39.4% had hepatitis B virus (HBV)-related cirrhosis [14]. Thus, serum myostatin can be applicable to predict clinical outcomes in patients with other etiologies of chronic HCV or HBV infection. Given that the major etiology of liver cirrhosis in western countries is alcoholic [20], it might be important to clarify prognostic performance of serum myostatin for clinical outcomes in patients with alcoholic cirrhosis. However, further studies in large cohorts with other etiologies are needed to validate the prognostic performance of serum myostatin levels.

We chose patients with alcoholic cirrhosis because previous studies reported that chronic alcohol consumption induced expression of myostatin in animal models and in patients with hypertensive heart disease [40,41]. Although many studies suggested that chronic alcohol drinking was associated with elevated serum myostatin levels, there were no concrete data regarding prognostic performance of serum myostatin and HCC development in patients with alcoholic cirrhosis. Particularly, in patients with NASH-related cirrhosis, there were no data regarding the association between serum myostatin and clinical outcomes. We guess that it may be difficult to collect serum samples from large cohorts with NASH-related cirrhosis because liver biopsy for accurate diagnosis of NASH-related cirrhosis is not usually performed in real clinical settings.

Second, no data regarding the estimation of sarcopenia and muscle dysfunction, such as a reduction in hand-grip strength, were identified in this study. The interactions between sarcopenia, muscle dysfunction, and myostatin and how they can affect HCC risk in cirrhotic patients are unknown. However, in this study, we looked at the associations of myostatin with more commonly used blood test variables and body mass indices (BMIs) for HCC risk; thus, we suggested a promising prognostic role for serum myostatin levels in HCC risk stratifications. Third, we could not determine a direct relationship between serum myostatin levels and liver fibrosis progression because no paired data of transient elastography or liver biopsy during follow-up periods were available in this study. However, considering that preclinical/clinical data have reported that myostatin and sarcopenia are associated with hepatic fibrosis [11,33,42,43], patients with high serum myostatin levels could have an increased risk of developing HCC because of progressing hepatic fibrosis. Fourth, although we excluded patients with active alcoholism in this study, objective evidence regarding alcohol abstinence is difficult to obtain because there are no objective measures of abstinence in the practical field. Given that the overall HCC incidence rates in this study were not higher than those in similar previous studies [19,29,44], we feel that patients with active alcoholism were excluded through a meticulous review of the medical records. Lastly, although serum des-gamma-carboxyl prothrombin and glypican-3 were important tumor markers for HCC development, the prognostic performance of the two markers was not considered in this study. Further study comparing the prognostic performance of serum myostatin with that of the two markers in cirrhotic patients should be performed.

## 5. Conclusions

In conclusion, the present multicenter cohort study found a significant association between serum myostatin levels and the risk of developing HCC in Asian ALC patients. Thus, serum myostatin levels could serve as prognostic markers to determine HCC risk in ALC patients, which might be helpful for the precise risk stratification in these patients. Further large studies are required to validate our results and evaluate the prognostic value of the serum myostatin levels in populations with different clinical backgrounds.

## Figures and Tables

**Table 1:** Baseline characteristics.

Variables	Total (*n* = 1077)	No HCC (*n* = 998)	HCC (*n* = 79)	*p*-Value ^a^	Low Myostatin (*n* = 781)	High Myostatin (*n* = 296)	*p*-Value ^a^
Age, years	55 (47, 62)	55 (47, 61)	57 (55, 63)	0.003	55 (46, 62)	55 (49, 59)	0.73
Male, n (%)	895 (83.1)	820 (82.2)	75 (94.9)	0.002	615 (78.7)	279 (94.3)	<0.001
Body mass index, kg/m^2^	23.0 (20.8, 25.3)	22.9 (20.8, 25.2)	24.1 (22.0, 26.9)	0.009	22.9 (20.8, 24.9)	23.7 (21.4, 26.2)	<0.001
Diabetes mellitus, n (%)	247 (22.9)	223 (22.3)	24 (30.4)	0.13	185 (23.7)	62 (20.9)	0.34
AST, IU/L	53 (38, 84)	53 (38, 85)	62 (37, 78)	0.60	53 (37, 88)	53 (38, 78)	0.97
ALT, IU/L	22 (13, 37)	22 (13, 37)	23 (16, 34)	0.43	23 (14, 39)	21 (12, 34)	0.02
Total bilirubin, mg/dL	1.7 (0.9, 3.6)	1.7 (0.9, 3.5)	1.9 (1.2, 3.1)	0.70	1.6 (0.8, 3.5)	1.9 (1.1, 4.0)	0.005
INR	1.3 (1.2, 1.7)	1.3 (1.2, 1.6)	1.4 (1.2, 1.6)	0.90	1.3 (1.1, 1.6)	1.4 (1.3, 1.7)	<0.001
Creatinine, mg/dL	0.9 (0.8, 1.2)	0.9 (0.7, 1.2)	0.9 (0.7, 1.1)	0.10	0.9 (0.7, 1.2)	1.0 (0.8, 1.2)	0.006
Platelets, × 10^9^/L	105 (71, 168)	107 (73, 174)	93 (69, 137)	0.03	113 (75, 183)	94 (63, 125)	<0.001
Albumin, g/dL	3.1 (2.6, 3.6)	3.1 (2.6, 3.6)	3.0 (2.6, 3.6)	0.998	3.1 (2.7, 3.7)	2.9 (2.6, 3.4)	<0.001
Toronto HCC risk index	236 (186, 255)	236 (186, 255)	255 (205, 286)	<0.001	225 (166, 255)	236 (205, 255)	<0.001
Low risk	88 (8.2)	86 (8.6)	2 (2.5)	<0.001	84 (10.8)	4 (1.4)	<0.001
Intermediate risk	650 (60.4)	613 (61.4)	37 (46.8)		489 (62.6)	161 (54.4)	
High risk	339 (31.5)	299 (30.0)	40 (50.6)		208 (26.6)	131 (44.3)	
FIB-4 index	6.5 (3.7, 11.3)	6.4 (3.6, 11.1)	8.0 (4.3, 11.8)	0.04	6.0 (3.2, 10.4)	7.6 (4.9, 12.3)	<0.001
MELD score	13.2 (9.5, 18.2)	13.3 (9.5, 18.6)	12.6 (10.2, 17.6)	0.29	12.7 (9.0, 17.7)	14.5 (11.0, 19.3)	<0.001
Child–Pugh class							
A, n (%)	343 (31.8)	318 (31.9)	25 (31.6)	0.53	274 (35.1)	69 (23.3)	0.001
B, n (%)	464 (43.1)	426 (42.7)	38 (48.1)		322 (41.2)	142 (48.0)	
C, n (%)	270 (25.1)	254 (25.5)	16 (20.3)		185 (23.7)	85 (28.7)	
α-fetoprotein	4.2 (2.8, 6.7)	4.2 (2.8, 6.7)	5.4 (3.5, 7.0)	0.053	4.1 (2.7, 6.5)	4.8 (3.1, 7.4)	0.003
Myostatin, ng/mL	3.3 (2.1, 5.2)	3.2 (2.0, 4.9)	6.1 (4.0, 8.9)	<0.001	2.6 (1.8, 3.6)	7.2 (6.0, 9.5)	<0.001

Values are expressed as the mean with standard deviation or median with interquartile range (IQR) for continuous variables and frequency with proportion for categorical variables. The total population was divided into two groups (a low myostatin group and a high myostatin group) using a cutoff point of 4.97 ng/mL, which provided the maximum sum of the specificity and sensitivity in being able to predict the risk of hepatocellular carcinoma (HCC) development within 5 years. ^a^ *p*-value estimated by χ^2^-test or Fisher’s exact test for categorical variables, and Mann–Whitney U test or Kruskal–Wallis test for continuous variables. Abbreviations: ALT, alanine aminotransferase; AST, aspartate aminotransferase; FIB-4, fibrosis-4; INR, international normalized ratio for prothrombin time; MELD, model for end-stage liver disease.

**Table 2:** Cox proportional hazards regression analysis for HCC development in total population (*n* = 1077).

Variables	Univariable		Multivariable	
	HR (95% CI)	*p*-Value ^a^	HR (95% CI)	*p*-Value ^a^
Age (per year increase)	1.04 (1.01–1.06)	<0.001	1.03 (1.01–1.06)	0.004
Gender (male vs. female)	4.05 (1.48–11.07)	0.006	2.79 (1.01–7.75)	0.04
Body mass index, kg/m^2^	1.08 (1.02–1.13)	0.006	1.05 (0.98–1.12)	0.12
Diabetes mellitus (yes vs. no)	1.42 (0.88–2.29)	0.16		
AST (per IU/L)	0.999 (0.996–1.001)	0.34		
ALT (per IU/L)	0.995 (0.988–1.003)	0.20		
Total bilirubin (per mg/dL)	0.97 (0.91–1.04)	0.39		
Albumin (per g/dL)	0.60 (0.43–0.84)	0.003	0.73 (0.50–1.10)	0.09
Platelets (per × 10^9^/L)	0.993 (0.989–0.996)	<0.001	0.996 (0.992–0.999)	0.03
INR	0.99 (0.89–1.11)	0.90		
Creatinine (per mg/dL)	0.90 (0.51–1.56)	0.69		
Child–Pugh score	1.09 (0.99–1.21)	0.09		
FIB-4	1.01 (0.99–1.03)	0.18		
MELD score	1.02 (0.98–1.10)	0.37		
Myostatin (per ng/mL)	1.24 (1.19–1.30)	<0.001	1.18 (1.12–1.24)	<0.001

^a^*p*-value estimated by Cox proportional hazard regression. Abbreviations: ALT, alanine aminotransferase; AST, aspartate aminotransferase; CI, confidence interval; FIB-4, fibrosis-4; HR, hazard ratio; INR, international normalized ratio for prothrombin time; MELD, model for end-stage liver disease.

## Supplementary Materials

The following are available online at https://www.mdpi.com/2072-6694/12/11/3347/s1, Figure S1: A proposed diagram of the possible mechanism of myostatin for HCC development, fibrosis, and hepatic inflammation.

## Abbreviations

ALC	alcoholic liver cirrhosis
CI	confidence interval
CP	Child–Pugh
HCC	hepatocellular carcinoma
HR	hazard ratio
MELD	model for end-stage liver disease
THRI	Toronto hepatocellular carcinoma risk index
