# Supplementary material for: Serum Myostatin Predicts the Risk of Hepatocellular Carcinoma in Patients with Alcoholic Cirrhosis: A Multicenter Study
Source: Cancers (Basel). 2020 Nov 12;12(11):3347. doi: 10.3390/cancers12113347 (PMC7697795; doi:10.3390/cancers12113347)
Supplement: Supplementary file 1 [file cancers-12-03347-s001.pdf]

cancers

MDPI

Supplementary Materials: Serum Myostatin Predicts the Risk of Hepatocellular Carcinoma in Patients with Alcoholic Cirrhosis: A Multicenter Study

Ji Hyun Kim, Seong Hee Kang, Minjong Lee, Gi Soo Youn, Tae Suk Kim, Baek Gyu Jun, Moon Young Kim, Young Don Kim, Gab Jin Cheon, Dong Joon Kim, Soon Koo Baik, Dae Hee Choi and Ki Tae Suk

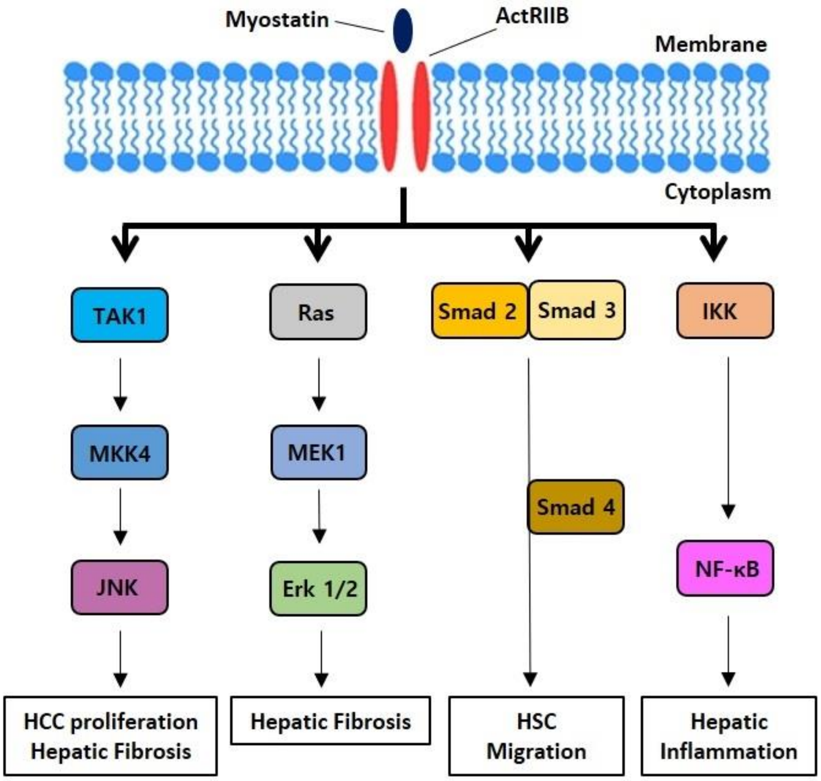

Figure S1. A proposed diagram of the possible mechanism of myostatin for HCC development, fibrosis, and hepatic inflammation. Myostatin can bind to ActRIIB in hepatic stellate cells that lead activation of JNK via signaling molecules consisting of MKK. Activated JNKs phosphorylate their substrates and mitochondrial proteins that induce various responses including HCC proliferation, fibrosis, and inflammation. Through activation of MEK, Erk 1/2 pathway has also been reported to participate in myostatin signal transduction and hepatic fibrogenesis. Activation of Smad complex results in migration of hepatic stellate cells, while IKK activates NF-κB that has been reported to increases expression of inflammation-related genes. Abbreviations: ActRIIB, type-2 activin receptor; HCC, hepatocellular carcinoma; HSC, hepatic stellate cell; IKK, IκB kinase; JNK, c-Jun-N-terminal kinase; MAP; mitogen-activated protein; MKK, mitogen-activated protein kinase kinase; NF-κB, nuclear factor-κB; TAK1, transforming growth factor β–activated kinase 1.
